# Omega-3 polyunsaturated fatty acid intake and pain, inflammatory cytokines, and quality of life in endometriosis

**DOI:** 10.3389/fnut.2026.1768244

**Published:** 2026-03-04

**Authors:** Ying Huang, Wen-Jing Zhou, Lin Zhu, Dong-Dong Ni

**Affiliations:** 1 Department of Gynecology and Obstetrics, The Ninth Medical Center, Chinese PLA General Hospital, Beijing, China; 2 Clinical Laboratory Center, The First Medical Center, Chinese PLA General Hospital, Beijing, China

**Keywords:** endometriosis, inflammatory cytokines, interleukin-6, omega-3 polyunsaturated fatty acid supplementation, pelvic pain, tumor necrosis factor alpha

## Abstract

**Background:**

Endometriosis is a chronic inflammatory gynecologic disorder associated with pelvic pain and impaired health-related quality of life (HRQoL). Omega-3 polyunsaturated fatty acids (PUFAs) have anti-inflammatory potential and may confer adjunctive benefit when combined with conventional therapy. This study evaluated the association between adjunctive omega-3 PUFA intake and pain, inflammatory biomarkers, and HRQoL in endometriosis.

**Methods:**

This retrospective, time-period–based cohort study included patients with confirmed endometriosis treated at a single center between January 2021 and December 2024. Conventional therapy during 2021–2022 served as the control period, whereas conventional therapy plus omega-3 PUFA supplementation during 2023–2024 constituted the exposure period. Outcomes included pain assessed by the visual analog scale (VAS), inflammatory biomarkers [interleukin-6 (IL-6), tumor necrosis factor alpha (TNF-α), and C-reactive protein (CRP)], and HRQoL measured using the 36-Item Short Form Health Survey (SF-36), including the Physical Component Summary (PCS) and Mental Component Summary (MCS). Multivariable logistic regression and inverse probability of treatment weighting (IPTW) were applied, with pre-specified subgroup and sensitivity analyses.

**Results:**

Among 302 screened patients, 289 were analyzed (control *n* = 138; omega-3 *n* = 151), with comparable baseline characteristics. Median exposure to eicosapentaenoic acid (EPA) plus docosahexaenoic acid (DHA) was 900 mg/day (interquartile range [IQR] 600–1,200) for 12 weeks (IQR 10–12). Compared with controls, the omega-3 group experienced greater reductions in overall pain (VAS Δ 3.0 ± 1.0 vs. 1.5 ± 0.9; *p* < 0.001), larger decreases in IL-6, TNF-α, and CRP (all *p* < 0.001), and greater improvements in SF-36 PCS (Δ 12.1 ± 5.2 vs. 5.3 ± 4.8) and MCS (Δ 10.3 ± 5.0 vs. 4.8 ± 4.6; both *p* < 0.001). Omega-3 PUFA intake was independently associated with clinically meaningful improvement, including VAS ≥2-point reduction (odds ratio [OR] 3.06, 95% confidence interval [CI] 1.85–5.06), PCS ≥5-point increase (OR 2.74, 95% CI 1.67–4.50), and MCS ≥5-point increase (OR 2.41, 95% CI 1.50–3.88), with consistent findings across subgroup, sensitivity, and IPTW analyses.

**Conclusions:**

Adjunctive omega-3 PUFA intake was associated with improved pain, reduced inflammatory biomarkers, and better HRQoL in endometriosis, warranting confirmation in future prospective randomized studies.

## 1 Introduction

Endometriosis is a chronic, estrogen-dependent inflammatory disorder characterized by ectopic endometrial-like tissue, commonly presenting with dysmenorrhea, chronic pelvic pain, and dyspareunia, and frequently accompanied by fatigue, gastrointestinal symptoms, and impaired fertility (1, 2). Despite advances in diagnostic strategies and therapeutic algorithms, symptom control remains suboptimal for a considerable proportion of patients, and recurrent or persistent pain continues to impose a sustained burden on daily functioning and psychosocial wellbeing. Recent clinical and translational syntheses increasingly conceptualize endometriosis as a multisystem condition in which immune dysregulation and inflammatory mediators contribute to lesion persistence, neuroangiogenesis, and pain sensitization (3, 4). Inflammatory cytokines are central to this framework. Elevated or dysregulated signaling of interleukin-6 (IL-6), tumor necrosis factor alpha (TNF-α), and other immune mediators has been repeatedly implicated in the pathophysiology of endometriosis, potentially linking systemic and local inflammatory milieus to symptom severity and disease progression (5). Contemporary narrative and mechanistic reviews emphasize that cytokine-driven interactions among immune cells, ectopic lesions, and surrounding pelvic tissues may amplify nociceptive pathways and sustain chronic pain states. These immunologic features provide a rationale for adjunct approaches that can modulate inflammation alongside conventional hormonal or analgesic strategies (6, 7).

Health-related quality of life (HRQoL) impairment is a core clinical outcome in endometriosis. Generic and disease-specific instruments consistently demonstrate reductions across physical, emotional, and social domains, with bodily pain often emerging as one of the most affected dimensions (8). Recent studies using 36-Item Short Form Health Survey (SF-36) and related measures have documented clinically meaningful decrements in both physical and mental components and have highlighted the long-term influence of pelvic pain on role functioning and psychological health (9, 10). Even when medical or surgical treatments improve symptoms, residual limitations in daily activities and emotional wellbeing remain common, reinforcing the need for comprehensive symptom-oriented strategies (11). Dietary factors and targeted nutritional interventions have therefore attracted increasing interest as potentially modifiable adjuncts in endometriosis management. Emerging literature on medical nutrition therapy and anti-inflammatory dietary patterns suggests plausible benefits through modulation of inflammatory tone, oxidative stress, and estrogen-related pathways, with fish-derived polyunsaturated fatty acids (PUFAs) representing a biologically relevant component of such approaches (12, 13). However, clinical evidence remains heterogeneous with respect to outcomes, assessment schedules, and integration with standard pharmacologic regimens, and real-world data that simultaneously address pain phenotypes, inflammatory biomarkers, and multidimensional QoL are still limited (14).

Against this background, the present study evaluated the association of adjunctive omega-3 PUFA intake with pain outcomes, inflammatory cytokine profiles, and SF-36–based QoL in patients with endometriosis receiving conventional therapy. By integrating symptom, laboratory, and patient-reported outcome measures within a unified analytic framework, this work aims to provide clinically informative evidence regarding the potential role of omega-3 PUFAs in multimodal endometriosis care.

## 2 Methods

### 2.1 Study design

This retrospective, time-period–based cohort study included patients with a confirmed diagnosis of endometriosis who received treatment at our institution between January 2021 and December 2024. Clinical data were extracted from electronic medical records and relevant follow-up documentation. To reflect changes in routine clinical practice over the study period, patients treated from January 2021 to December 2022 were managed with standard conventional therapy and were categorized as the control group, whereas patients treated from January 2023 to December 2024 received conventional therapy plus omega-3 PUFA intake as part of the treatment strategy and were categorized as the observation group. Group allocation was therefore determined by the calendar period of care rather than individual randomization. Informed consent was obtained from all participants. The study was approved by the hospital's ethics committee and conducted in accordance with relevant guidelines and the Declaration of Helsinki. Personal identifiers were removed to ensure data confidentiality and participant privacy.

### 2.2 Inclusion criteria and exclusion criteria

Patients were included if they: 1) Had a confirmed diagnosis of endometriosis documented in medical records (surgical/pathological and/or imaging-supported diagnosis); 2) Were treated at our institution between January 2021 and December 2024; 3) Received conventional therapy during the study period, with time-period allocation: January 2021–December 2022 as the control group and January 2023–December 2024 as the omega-3 PUFA plus conventional therapy group; 4) Had complete baseline demographic and clinical information; 5) Had available baseline and follow-up assessments of pain and at least one evaluation of inflammatory cytokines and QoL using validated instruments.

Patients were excluded if they: 1) Had major comorbidities that could substantially confound pain, inflammatory cytokines, or quality-of-life outcomes (e.g., active pelvic inflammatory disease, autoimmune or systemic inflammatory disorders, or malignancy); 2) Were pregnant or lactating during the primary evaluation window; 3) Had severe organ dysfunction that could affect inflammatory status or treatment comparability; 4) Had unclear or non-standardized omega-3 PUFA exposure (e.g., long-term self-initiated supplementation before baseline or insufficient documentation for accurate classification).

### 2.3 Treatment protocols

Control group (Conventional therapy): patients treated between January 2021 and December 2022 received standard conventional management for endometriosis. The core regimen consisted of symptom control and disease-suppressive therapy based on individual clinical presentation. Analgesic treatment primarily included non-steroidal anti-inflammatory drugs (NSAIDs) for pelvic pain and dysmenorrhea. Hormonal therapy was administered when clinically indicated, including combined oral contraceptives and/or progestin-based regimens, with gonadotropin-releasing hormone (GnRH) analog-based therapy considered for patients with refractory symptoms. Supportive measures (e.g., counseling regarding lifestyle and pain self-management) were provided as part of routine care. Treatment selection and duration were determined by attending physicians according to standard institutional practice, and key medication exposure was documented in the electronic medical record.

Observation group (Conventional therapy + omega-3 PUFA intake): patients treated between January 2023 and December 2024 received the same conventional management protocol as the control group, with the addition of omega-3 PUFA supplementation as an adjunctive strategy. Omega-3 PUFA supplementation was prescribed in a standardized oral fish oil–derived preparation containing eicosapentaenoic acid (EPA) and docosahexaenoic acid (DHA) that was routinely used at our institution during this phase. According to the institutional formulary/drug dictionary, the primary preparation provided 180 mg EPA and 120 mg DHA per capsule (total 300 mg EPA+DHA per capsule). The typical prescribed regimen was two to four capsules per day (corresponding to a total EPA+DHA dose of approximately 600–1,200 mg/day, based on prescription records), initiated at the start of the treatment course (baseline) and intended to be continued throughout the routine follow-up period. Exposure to omega-3 PUFA was defined *a priori* based on documented prescription and/or pharmacy dispensing records indicating planned intake during the observation period. When available, continued use during follow-up was assessed from repeat prescriptions, dispensing records, and follow-up documentation. Patients with unclear exposure documentation precluding reliable classification, or with long-term self-initiated omega-3 supplementation before baseline, were excluded according to the pre-defined criteria. Analgesic therapy with non-steroidal anti-inflammatory drugs (NSAIDs) was administered on an as-needed basis according to routine clinical practice and symptom severity. Specific NSAID agents and doses were individualized and therefore not standardized; however, overall NSAID use was comparable between groups and was adjusted for in multivariable analyses.

### 2.4 Data collection

Clinical data were retrospectively collected from the electronic medical record system and relevant outpatient follow-up documentation for patients with endometriosis treated at our institution between January 2021 and December 2024. Data abstraction was performed using a standardized case report form by trained investigators. Discrepancies were resolved through re-review of source records. Baseline variables included demographic characteristics (age, body mass index, smoking status, and alcohol use), disease-related characteristics (disease duration and endometriosis stage), and treatment-related information. Endometriosis stage was recorded based on available surgical or imaging documentation as documented in the medical records. Conventional treatment exposure was extracted, including the use of non-steroidal anti-inflammatory drugs and hormonal therapy, with details of regimen type and timing when available. Omega-3 PUFA exposure was defined according to medical records indicating planned or documented omega-3 PUFA intake as part of the institutional treatment strategy during January 2023 to December 2024. For each eligible patient, the form, initiation timing within the treatment course, and documentation of continued use during follow-up were recorded when available. Patients treated during January 2021 to December 2022 received conventional therapy alone and constituted the control group.

Outcome data were collected at two pre-specified time points based on routine clinical practice: baseline assessment at the initiation of the treatment course and post-treatment assessment at the end of the defined evaluation window documented in the records. Pain was evaluated using the visual analog scale (VAS, 0–10), including overall pain and, when available, domain-specific measures for dysmenorrhea, chronic pelvic pain, and dyspareunia. The “overall pain” outcome was operationally defined as pelvic pain attributable to endometriosis and was derived from clinician-documented VAS assessments reflecting endometriosis-associated pain domains, specifically dysmenorrhea, chronic pelvic pain, and dyspareunia. HRQoL was assessed using the SF-36, with extraction of Physical Component Summary (PCS), Mental Component Summary (MCS), and domain scores, including bodily pain, mental health, social functioning, physical functioning, role physical, and role emotional. Inflammatory status was evaluated using routinely obtained laboratory tests. Serum levels of IL-6, TNF-α, and C-reactive protein (CRP) were collected at baseline and post-treatment when paired measurements were available. If multiple results were recorded within a single assessment window, the value closest to the corresponding clinical evaluation date was used. To support adjusted analyses and reduce confounding, additional covariates were extracted, including baseline VAS, baseline PCS/MCS, and key comorbidities documented in the medical history. Data completeness was assessed prior to analysis; patients lacking core paired outcome measures for pain, inflammatory cytokines, or SF-36 were excluded according to pre-defined criteria.

Inflammatory biomarkers were assessed using peripheral venous blood samples collected as part of routine clinical care. Serum IL-6, TNF-α, and C-reactive protein (CRP) levels were measured by the hospital's central laboratory, which follows standardized procedures for each biomarker. IL-6 and TNF-α concentrations were quantified using immunoassay methods, specifically enzyme-linked immunosorbent assay (ELISA) or chemiluminescent immunoassay (CLIA), as per manufacturer protocols and internal quality control standards. CRP was measured using a high-sensitivity immunoturbidimetric assay or an equivalent method. All biomarker results were recorded in the laboratory information system, and data were retrospectively extracted for analysis. If multiple measurements were available within the same assessment window, the value closest to the corresponding clinical evaluation date was used.

### 2.5 Statistical analysis

All statistical analyses were performed using IBM SPSS Statistics, version 27.0 (IBM Corp., Armonk, NY, USA). Continuous variables are presented as mean ± standard deviation, and categorical variables as number (percentage). Baseline characteristics between the control and observation groups were compared using the independent-samples *t*-test for continuous variables and the χ^2^ test for categorical variables. For outcome analyses, paired-samples *t*-tests were used to evaluate within-group changes in pain scores, inflammatory cytokines, and SF-36 indices from baseline to post-treatment. Between-group differences in post-treatment values and change scores (Δ) were assessed using independent-samples *t*-tests. Clinically meaningful improvement was defined *a priori* as overall pain reduction of ≥2 points on the VAS, and SF-36 improvement of ≥5 points for PCS and MCS.

To identify independent associations with clinically meaningful outcomes, multivariable logistic regression models were constructed to estimate odds ratios (ORs) and 95% confidence intervals (CIs). Covariates included demographic factors, disease-related variables, baseline severity measures, and conventional treatment exposure as recorded in the dataset. Subgroup analyses were performed stratified by disease stage (I–II vs. III–IV) and by hormonal therapy use (yes vs. no), and interaction terms were introduced to test for effect modification. Sensitivity analyses were conducted to assess robustness of the primary findings, including complete-case analyses, exclusion of patients with potential key confounders, additional adjustment for smoking and alcohol status, and restriction analyses excluding advanced-stage cases where applicable. Smoking status and alcohol consumption (current use: yes/no at baseline) were additionally adjusted for in sensitivity analyses. A propensity score–based inverse probability of treatment weighting (IPTW) approach was further applied as an alternative adjustment strategy. All tests were two-sided, and *p* < 0.05 was considered statistically significant.

## 3 Results

### 3.1 Patient enrollment

During the study period, 302 patients with endometriosis were assessed for eligibility. Thirteen patients were excluded based on the pre-defined criteria, including incomplete key outcome data for pain, inflammatory cytokines, or QoL (*n* = 6), unclear documentation of omega-3 PUFA exposure that precluded reliable group classification (*n* = 3), major comorbid inflammatory conditions likely to confound cytokine profiles or pain assessment (*n* = 2), pregnancy or lactation during the evaluation window (*n* = 1), and insufficient follow-up records for standardized outcome assessment (*n* = 1). Ultimately, 289 patients were included in the final analysis, comprising 138 patients in the control group and 151 patients in the observation group.

### 3.2 Summary of baseline comparability

In this retrospective cohort, baseline demographic and clinical variables were comparable between groups. The control group (*n* = 138) and the observation group (*n* = 151) showed no significant differences in age (32.8 ± 6.4 vs. 33.1 ± 6.1 years, *t* = −0.41, *p* = 0.684) or BMI (22.5 ± 2.8 vs. 22.7 ± 2.9 kg/m^2^, *t* = −0.60, *p* = 0.551). The prevalence of current smoking (10.1% vs. 10.6%, χ^2^ = 0.02, *p* = 0.900) and current alcohol use (15.9% vs. 17.2%, χ^2^ = 0.08, *p* = 0.771) was similar across groups. Disease duration did not differ significantly (4.2 ± 2.1 vs. 4.1 ± 2.0 years, *t* = 0.41, *p* = 0.679). The distribution of endometriosis stages was well balanced (χ^2^ = 0.04, *p* = 0.998). Key symptom profiles, including pelvic pain, dysmenorrhea, and dyspareunia, were also comparable (all *p* > 0.80). Regarding conventional treatment exposure, the proportions of NSAID use and hormonal therapy use were similar between groups (*p* = 0.750 and *p* = 0.809, respectively; Table 1).

**Table 1:** Baseline demographic and clinical characteristics of the study population.

**Variable**	**Control group (*n* = 138)**	**Observation group (*n* = 151)**	**Test statistic**	***p*-value**
Age, years	32.8 ± 6.4	33.1 ± 6.1	*t* = −0.41	0.684
BMI, kg/m^2^	22.5 ± 2.8	22.7 ± 2.9	*t* = −0.60	0.551
Current smoking, *n* (%)	14 (10.1)	16 (10.6)	χ^2^ = 0.02	0.900
Current alcohol use, *n* (%)	22 (15.9)	26 (17.2)	χ^2^ = 0.08	0.771
Disease duration, years	4.2 ± 2.1	4.1 ± 2.0	*t* = 0.41	0.679
**Endometriosis stage**, ***n*** **(%)**				
Stage I	32 (23.2)	36 (23.8)	χ^2^ = 0.04	0.998
Stage II	46 (33.3)	51 (33.8)		
Stage III	40 (29.0)	43 (28.5)		
Stage IV	20 (14.5)	21 (13.9)		
Pelvic pain, *n* (%)	98 (71.0)	108 (71.5)	χ^2^ = 0.01	0.924
Dysmenorrhea, *n* (%)	112 (81.2)	123 (81.5)	χ^2^ = 0.00	0.948
Dyspareunia, *n* (%)	54 (39.1)	61 (40.4)	χ^2^ = 0.05	0.826
NSAID use, *n* (%)	101 (73.2)	113 (74.8)	χ^2^ = 0.10	0.750
Hormonal therapy use, *n* (%)	84 (60.9)	94 (62.3)	χ^2^ = 0.06	0.809

BMI, body mass index; NSAID, non-steroidal anti-inflammatory drug.

### 3.3 Omega-3 PUFA exposure characteristics

Among patients in the observation group (*n* = 151), omega-3 PUFA supplementation was prescribed pre-dominantly as a standard fish oil capsule containing 180 mg EPA and 120 mg DHA per capsule (300 mg EPA+DHA); a minority received an alternative formulation with comparable EPA/DHA content according to the institutional formulary. The median prescribed daily dose of combined EPA and DHA was 900 mg/day (IQR: 600–1,200 mg/day; range: 300–1,800 mg/day), corresponding to a median of 3 capsules/day (IQR: 2–4; range: 1–6). The median duration of omega-3 PUFA exposure from baseline to the post-treatment assessment was 12 weeks (IQR: 10–12 weeks; range: 8–16 weeks). Detailed exposure characteristics, including formulation-specific EPA/DHA content, prescribed dose distributions, and exposure duration, are provided in Supplementary Table S1.

### 3.4 Pain outcomes

Across the study cohort, both groups demonstrated significant within-group reductions in overall pain after treatment. In the control group, the overall VAS score decreased from 6.7 ± 1.2 to 5.2 ± 1.5 (Δ = 1.5 ± 0.9; *t* = 20.37, *p* < 0.001). In the observation group receiving conventional therapy plus omega-3 PUFA intake, the overall VAS score declined from 6.6 ± 1.2 to 3.6 ± 1.6 (Δ = 3.0 ± 1.0; *t* = 36.42, *p* < 0.001). Between-group comparisons indicated lower post-treatment pain scores in the observation group than in the control group (*t* = 8.85, *p* < 0.001), accompanied by a larger magnitude of pain improvement (*t* = −13.91, *p* < 0.001). Analyses of specific pain domains yielded consistent patterns. Dysmenorrhea, chronic pelvic pain, and dyspareunia all improved significantly within each group; however, the observation group exhibited greater reductions across domains, reflected in both lower post-treatment scores and larger Δ values (all *p* < 0.001). These findings support a potential adjunctive benefit of omega-3 PUFA intake in alleviating multiple pain dimensions of endometriosis when added to conventional therapy (Table 2).

**Table 2:** Pain outcomes (overall and domain-specific) before and after treatment.

**Pain outcome (VAS 0–10)**	**Control baseline (*n* = 138)**	**Control post**	**Control Δ**	**Observation baseline (*n* = 151)**	**Observation post**	**Observation Δ**	**Within-group change (control)**	**Within-group change (observation)**	**Between-group (post)**	**Between-group (Δ)**
Overall pain	6.7 ± 1.2	5.2 ± 1.5	1.5 ± 0.9	6.6 ± 1.2	3.6 ± 1.6	3.0 ± 1.0	*t* = 20.37, *p* < 0.001	*t* = 36.42, *p* < 0.001	*t* = 8.85, *p* < 0.001	*t* = −13.91, *p* < 0.001
Dysmenorrhea	7.0 ± 1.3	5.5 ± 1.5	1.5 ± 0.9	7.2 ± 1.2	4.0 ± 1.4	3.2 ± 1.0	*t* = 21.01, *p* < 0.001	*t* = 39.58, *p* < 0.001	*t* = 8.47, *p* < 0.001	*t* = −15.21, *p* < 0.001
Chronic pelvic pain	6.3 ± 1.4	5.1 ± 1.6	1.2 ± 0.8	6.4 ± 1.2	3.8 ± 1.6	2.6 ± 1.0	*t* = 17.70, *p* < 0.001	*t* = 32.95, *p* < 0.001	*t* = 7.00, *p* < 0.001	*t* = −13.43, *p* < 0.001
Dyspareunia	5.8 ± 1.3	4.7 ± 1.6	1.1 ± 0.7	6.0 ± 1.3	3.8 ± 1.6	2.1 ± 0.9	*t* = 18.13, *p* < 0.001	*t* = 29.70, *p* < 0.001	*t* = 4.40, *p* < 0.001	*t* = −10.60, *p* < 0.001

VAS, visual analog scale; Δ, change from baseline to post-treatment.

### 3.5 Inflammatory cytokine outcomes

At baseline, inflammatory cytokine levels were comparable between groups. There were no significant differences in IL-6 (9.8 ± 3.0 vs. 9.7 ± 3.1 pg/ml, *t* = 0.28, *p* = 0.781), TNF-α (15.2 ± 4.2 vs. 15.0 ± 4.1 pg/ml, *t* = 0.41, *p* = 0.683), or CRP (4.6 ± 1.9 vs. 4.5 ± 1.8 mg/L, *t* = 0.46, *p* = 0.647). After treatment, the observation group receiving conventional therapy plus omega-3 PUFA intake showed lower post-treatment levels of IL-6, TNF-α, and CRP compared with the control group (all *p* < 0.001). Consistently, the magnitude of reduction (Δ) in each marker was greater in the observation group than in the control group (IL-6: 3.9 ± 1.8 vs. 1.8 ± 1.5; TNF-α: 4.2 ± 1.9 vs. 1.9 ± 1.6; CRP: 1.9 ± 1.1 vs. 0.8 ± 0.9; all *p* < 0.001; Table 3).

**Table 3:** Inflammatory cytokine levels at baseline and after treatment.

**Cytokine**	**Control baseline**	**Control post**	**Control Δ**	**Observation baseline**	**Observation post**	**Observation Δ**	***p*-value (baseline/post/Δ)**
IL-6, pg/ml	9.8 ± 3.0	8.0 ± 2.8	1.8 ± 1.5	9.7 ± 3.1	5.9 ± 2.6	3.9 ± 1.8	0.781/ < 0.001/ < 0.001
TNF-α, pg/ml	15.2 ± 4.2	13.3 ± 3.9	1.9 ± 1.6	15.0 ± 4.1	10.8 ± 3.6	4.2 ± 1.9	0.683/ < 0.001/ < 0.001
CRP, mg/L	4.6 ± 1.9	3.8 ± 1.7	0.8 ± 0.9	4.5 ± 1.8	2.6 ± 1.4	1.9 ± 1.1	0.647/ < 0.001/ < 0.001

IL-6, interleukin-6; TNF-α, tumor necrosis factor alpha; CRP, C-reactive protein; Δ, change from baseline to post-treatment.

### 3.6 Quality of life outcomes

Baseline SF-36 indices were comparable between groups, including PCS (*t* = −0.28, *p* = 0.777) and MCS (*t* = −0.27, *p* = 0.786). After treatment, both groups exhibited significant within-group improvements in overall QoL. The control group showed increases in PCS and MCS (PCS Δ = 5.3 ± 4.8, paired *t* = 12.97, *p* < 0.001; MCS Δ = 4.8 ± 4.6, paired *t* = 12.26, *p* < 0.001). The observation group demonstrated larger gains (PCS Δ = 12.1 ± 5.2, paired *t* = 28.59, *p* < 0.001; MCS Δ = 10.3 ± 5.0, paired *t* = 25.31, *p* < 0.001). Between-group comparisons indicated higher post-treatment PCS and MCS scores in the observation group (PCS: *t* = −6.77, *p* < 0.001; MCS: *t* = −5.38, *p* < 0.001), accompanied by greater improvement magnitudes (PCS Δ: *t* = −11.56, *p* < 0.001; MCS Δ: *t* = −9.74, *p* < 0.001). Domain-specific analyses further supported this pattern. Pain-related QoL improved in both groups, with a larger increase in BP in the observation group (Δ = 23.0 ± 9.0 vs. 9.5 ± 8.0, *p* < 0.001). Emotional/psychological and social functioning domains also showed greater enhancement in the observation group, including MH and SF (both Δ*p* < 0.001). Functional dimensions reflecting daily activities and role limitations (PF, RP, and RE) improved more substantially in the observation group than in the control group (all post-treatment and Δ*p* < 0.001; Table 4).

**Table 4:** SF-36 overall and domain-specific quality-of-life outcomes.

**SF-36 outcome (0–100)**	**Control baseline (*n* = 138)**	**Control post**	**Control Δ**	**Observation baseline (*n* = 151)**	**Observation post**	**Observation Δ**	***p*-value (baseline/post/Δ)**
Physical component summary (PCS)	44.8 ± 8.9	50.1 ± 9.0	5.3 ± 4.8	45.1 ± 9.1	57.2 ± 8.8	12.1 ± 5.2	0.777/ < 0.001/ < 0.001
Mental component summary (MCS)	46.2 ± 9.5	51.0 ± 9.3	4.8 ± 4.6	46.5 ± 9.2	56.8 ± 9.0	10.3 ± 5.0	0.786/ < 0.001/ < 0.001
Bodily pain (BP)	38.5 ± 12.0	48.0 ± 12.5	9.5 ± 8.0	39.0 ± 11.5	62.0 ± 12.0	23.0 ± 9.0	0.718/ < 0.001/ < 0.001
Mental health (MH)	44.0 ± 11.0	50.5 ± 10.5	6.5 ± 6.0	44.2 ± 10.8	58.5 ± 10.2	14.3 ± 6.5	0.876/ < 0.001/ < 0.001
Social functioning (SF)	40.0 ± 13.0	47.5 ± 12.5	7.5 ± 7.0	40.5 ± 12.8	57.5 ± 12.0	17.0 ± 7.5	0.742/ < 0.001/ < 0.001
Physical functioning (PF)	55.0 ± 15.0	61.0 ± 14.8	6.0 ± 6.5	55.5 ± 14.5	69.5 ± 14.0	14.0 ± 7.0	0.774/ < 0.001/ < 0.001
Role physical (RP)	42.0 ± 18.0	50.0 ± 17.0	8.0 ± 9.0	42.5 ± 17.5	63.0 ± 16.5	20.5 ± 9.5	0.811/ < 0.001/ < 0.001
Role emotional (RE)	45.0 ± 17.0	52.5 ± 16.5	7.5 ± 8.5	45.2 ± 16.8	62.5 ± 16.0	17.3 ± 9.0	0.920/ < 0.001/ < 0.001
Vitality (VT)	41.0 ± 10.5	47.0 ± 10.0	6.0 ± 5.5	41.3 ± 10.2	55.2 ± 9.8	13.9 ± 6.0	0.806/ < 0.001/ < 0.001

SF-36, 36-item short form health survey; PCS, physical component summary; MCS, mental component summary; BP, bodily pain; MH, mental health; SF, social functioning; PF, physical functioning; RP, role physical; RE, role emotional; VT, vitality; Δ, change from baseline to post-treatment.

Higher scores indicate better quality of life.

### 3.7 Multivariable logistic regression analysis of pain and quality of life improvement

In the adjusted logistic model, adjunctive omega-3 PUFA intake was independently associated with a higher likelihood of clinically meaningful overall pain improvement (ΔVAS ≥2) compared with conventional therapy alone (OR = 3.06, 95% CI 1.85–5.06, *p* < 0.001). Higher baseline pain severity was also a significant predictor of achieving this level of improvement (OR = 1.58 per 1-point increase in baseline VAS, 95% CI 1.29–1.94, *p* < 0.001). In contrast, longer disease duration was associated with a reduced probability of substantial pain relief (OR = 0.90 per year, 95% CI 0.84–0.97, *p* = 0.006). Age, BMI, disease stage, and concomitant conventional treatment exposure were not independently associated with the pain improvement endpoint in this model. For quality-of-life outcomes, omega-3 PUFA intake also remained an independent factor associated with clinically meaningful gains in SF-36 components. Patients in the observation group were more likely to achieve ΔPCS ≥5 (OR = 2.74, 95% CI 1.67–4.50, *p* < 0.001) and ΔMCS ≥5 (OR = 2.41, 95% CI 1.50–3.88, *p* < 0.001). Higher baseline PCS and MCS scores were inversely associated with achieving clinically meaningful improvement in the corresponding domains (both *p* < 0.001), whereas longer disease duration showed modest negative associations with both PCS and MCS improvement thresholds (Table 5).

**Table 5:** Multivariable analyses for pain and QoL improvement.

**Variable**	**Clinically meaningful pain improvement (ΔVAS ≥2) OR (95% CI)**	***p*-value**	**Clinically meaningful PCS improvement (ΔPCS ≥5) OR (95% CI)**	***p*-value**	**Clinically meaningful MCS improvement (ΔMCS ≥5) OR (95% CI)**	***p*-value**
Omega-3 PUFA group (vs. control)	3.06 (1.85–5.06)	< 0.001	2.74 (1.67–4.50)	< 0.001	2.41 (1.50–3.88)	< 0.001
Age, per year	0.99 (0.96–1.02)	0.462	0.98 (0.96–1.01)	0.201	0.99 (0.96–1.01)	0.352
BMI, per kg/m^2^	0.97 (0.91–1.04)	0.405	0.96 (0.90–1.02)	0.176	0.97 (0.91–1.03)	0.327
Disease duration, per year	0.90 (0.84–0.97)	0.006	0.92 (0.86–0.99)	0.020	0.93 (0.87–1.00)	0.047
Stage III–IV (vs. I–II)	0.74 (0.46–1.20)	0.224	0.68 (0.42–1.10)	0.114	0.72 (0.45–1.16)	0.182
Baseline overall VAS, per 1-point	1.58 (1.29–1.94)	< 0.001	—	—	—	—
Baseline PCS, per 1-point	—	—	0.93 (0.91–0.96)	< 0.001	—	—
Baseline MCS, per 1-point	—	—	—	—	0.94 (0.92–0.96)	< 0.001
NSAID use (yes vs. no)	1.16 (0.68–1.99)	0.587	1.12 (0.66–1.90)	0.682	1.08 (0.65–1.78)	0.776
Hormonal therapy (yes vs. no)	1.31 (0.82–2.10)	0.259	1.38 (0.87–2.19)	0.169	1.29 (0.82–2.04)	0.272

VAS, visual analog scale; SF-36, 36-item short form health survey; PCS, physical component summary; MCS, mental component summary; BMI, body mass index; NSAID, non-steroidal anti-inflammatory drug; PUFA, polyunsaturated fatty acid; Δ, change from baseline to post-treatment.

### 3.8 Subgroup and sensitivity analyses

Subgroup analyses demonstrated a consistent association between adjunctive omega-3 PUFA intake and clinically meaningful improvements in both pain and physical QoL. The odds of achieving significant pain reduction (ΔVAS ≥2) were higher in the omega-3 PUFA group across disease severity strata, including Stage I–II (OR = 3.22, 95% CI 1.75–5.94) and Stage III–IV (OR = 2.84, 95% CI 1.42–5.67), with no evidence of effect modification by stage (*p* for interaction = 0.741). Similar consistency was observed for QoL improvement (ΔPCS ≥5) in Stage I–II (OR = 2.81, 95% CI 1.54–5.14) and Stage III–IV (OR = 2.61, 95% CI 1.31–5.20). When stratified by hormonal therapy use, omega-3 PUFA intake remained associated with increased odds of clinically meaningful pain and PCS improvement both among patients receiving hormonal therapy (pain: OR = 2.97, 95% CI 1.60–5.49; PCS: OR = 2.66, 95% CI 1.46–4.85) and those not receiving hormonal therapy (pain: OR = 3.18, 95% CI 1.55–6.53; PCS: OR = 2.88, 95% CI 1.38–6.00), with no significant interaction (*p* for interaction = 0.689; Table 6).

**Table 6:** Subgroup analyses for the association of omega-3 PUFA intake with clinically meaningful improvement.

**Subgroup**	***n***	**Pain improvement (ΔVAS ≥2) OR (95% CI)**	***p*-value**	**QoL improvement (ΔPCS ≥5) OR (95% CI)**	***p*-value**	***p* for interaction**
Stage I–II	165	3.22 (1.75–5.94)	<0.001	2.81 (1.54–5.14)	<0.001	0.741
Stage III–IV	124	2.84 (1.42–5.67)	0.003	2.61 (1.31–5.20)	0.006	
With hormonal therapy	178	2.97 (1.60–5.49)	<0.001	2.66 (1.46–4.85)	<0.001	0.689
Without hormonal therapy	111	3.18 (1.55–6.53)	0.002	2.88 (1.38–6.00)	0.005	

OR, odds ratio; CI, confidence interval; VAS, visual analog scale; SF-36, 36-item short form health survey; PCS, physical component summary; PUFA, polyunsaturated fatty acid; Δ, change from baseline to post-treatment.

Sensitivity analyses further supported the robustness of the primary findings. The association of omega-3 PUFA intake with clinically meaningful pain improvement and PCS improvement remained stable in the complete-case analysis (pain: OR = 2.98; PCS: OR = 2.69), after excluding patients with potential inflammatory comorbidities (pain: OR = 3.12; PCS: OR = 2.81), with additional adjustment for smoking and alcohol status (pain: OR = 2.95; PCS: OR = 2.63), after excluding Stage IV disease (pain: OR = 3.21; PCS: OR = 2.88), and in the IPTW model (pain: OR = 2.90; PCS: OR = 2.58). Results remained consistent after additional adjustment for smoking and alcohol use (Table 7).

**Table 7:** Sensitivity analyses for the association of omega-3 PUFA intake with clinically meaningful improvement.

**Sensitivity analysis scenario**	**Pain improvement (ΔVAS ≥2) OR (95% CI)**	***p*-value**	**QoL improvement (ΔPCS ≥5) OR (95% CI)**	***p*-value**
Main adjusted model^*^	3.06 (1.85–5.06)	<0.001	2.74 (1.67–4.50)	<0.001
Complete-case analysis	2.98 (1.76–5.03)	< 0.001	2.69 (1.61–4.49)	<0.001
Excluding inflammatory comorbidities	3.12 (1.86–5.24)	< 0.001	2.81 (1.69–4.67)	<0.001
Additional adjustment for smoking/alcohol	2.95 (1.78–4.91)	<0.001	2.63 (1.58–4.38)	<0.001
Excluding Stage IV disease	3.21 (1.89–5.46)	<0.001	2.88 (1.71–4.84)	<0.001
IPTW model^**^	2.90 (1.74–4.84)	<0.001	2.58 (1.55–4.31)	< 0.001

^*^Main adjusted model covariates (example): age, BMI, disease duration, stage, baseline VAS/PCS, NSAID use, hormonal therapy.

^**^IPTW = inverse probability of treatment weighting.

OR, odds ratio; CI, confidence interval; VAS, visual analog scale; SF-36, 36-item short form health survey; PCS, physical component summary; BMI, body mass index; NSAID, non-steroidal anti-inflammatory drug; PUFA, polyunsaturated fatty acid; IPTW, inverse probability of treatment weighting.

## 4 Discussion

This retrospective, time-period–based cohort study examined whether adjunctive omega-3 PUFA supplementation was associated with improved pain, inflammatory biomarkers, and HRQoL among patients with endometriosis receiving conventional therapy in routine practice. Overall, the findings were consistent across complementary outcome domains. Although both groups demonstrated significant within-group reductions in overall pelvic pain and in endometriosis-associated pain domains, patients receiving omega-3 PUFA achieved larger absolute improvements in overall VAS as well as dysmenorrhea, chronic pelvic pain, and dyspareunia. In parallel, systemic inflammatory activity decreased more substantially in the omega-3 PUFA group, as reflected by greater reductions in IL-6, TNF-α, and CRP, and HRQoL gains were broader and larger, spanning SF-36 physical and mental component summaries and multiple function- and pain-related subscales. These associations remained robust after adjustment for baseline severity, disease duration, endometriosis stage, and concomitant conventional treatments, and were further supported by subgroup analyses, sensitivity analyses (including additional adjustment for smoking and alcohol use), and IPTW. A key contribution of this work is the integrated evaluation linking symptom outcomes, inflammatory profiling, and multidimensional HRQoL within a single analytic framework, together with transparent characterization of omega-3 PUFA exposure, including EPA/DHA composition, prescribed dose range, and duration. From a clinical perspective, our findings suggest that omega-3 PUFA supplementation may be considered as a pragmatic adjunct to conventional management in symptomatic endometriosis, particularly for patients with persistent pelvic pain or inflammatory profiles that remain elevated despite standard therapy. The consistent benefits observed across dysmenorrhea, chronic pelvic pain, and dyspareunia indicate potential utility for multidimensional pain control and for improving patient-reported functioning. These results support the integration of basic nutritional assessment into routine endometriosis care and reinforce the role of anti-inflammatory dietary counseling within multidisciplinary management programs. Importantly, supplementation decisions should remain individualized, with careful consideration of formulation (EPA/DHA composition), dose, tolerability, and potential interactions with concomitant analgesic or hormonal therapies. Given the retrospective design, prospective studies incorporating standardized dietary assessment and protocolized co-interventions are warranted to confirm causality and to refine patient selection and dosing strategies.

Enrollment in this cohort was near-complete, with few exclusions primarily attributable to missing core outcomes or unverifiable omega-3 PUFA exposure, resulting in two groups that were well balanced at baseline with respect to demographics, disease duration, endometriosis stage, symptom profiles, and concomitant NSAID and hormonal therapy use. This baseline comparability reduces, although does not eliminate, the likelihood that observed between-group differences were driven by pre-existing imbalance. Exposure characterization further supports interpretability of the findings: omega-3 PUFA supplementation was delivered with a clearly defined EPA/DHA composition, a clinically plausible dosing range (median combined EPA+DHA 900 mg/day), and a relatively uniform short-term follow-up (median 12 weeks). While both groups experienced symptomatic improvement consistent with expected effects of standard analgesic and hormonal management, the magnitude of pain reduction in the omega-3 PUFA group exceeded the pre-specified threshold for clinically meaningful improvement (ΔVAS ≥2). In adjusted analyses, omega-3 PUFA intake was independently associated with a substantially higher likelihood of achieving this endpoint, whereas longer disease duration was inversely associated with pain and HRQoL gains, suggesting that earlier disease stages may be more responsive to adjunctive anti-inflammatory strategies (15).

Biomarker findings were directionally concordant with the clinical signal. Although baseline IL-6, TNF-α, and CRP were similar between groups, post-treatment levels and reductions were significantly greater with omega-3 supplementation, supporting an association with attenuation of systemic inflammatory markers (16). While these assays do not establish lesion-level biology and may be influenced by unmeasured factors, the parallel improvement across pain, cytokines, and HRQoL strengthens internal coherence and biological plausibility of an anti-inflammatory contribution (17). Quality-of-life outcomes improved in both groups, but the omega-3 group exhibited larger gains in both PCS and MCS, as well as multiple domains spanning pain, emotional wellbeing, social functioning, and role limitations. The adjusted analyses similarly supported an independent association between omega-3 intake and clinically meaningful improvements in PCS and MCS (ORs 2.74 and 2.41, respectively), with expected ceiling effects reflected by inverse associations of baseline PCS/MCS with achieving pre-specified improvement thresholds. Subgroup analyses suggested that the association of omega-3 supplementation with clinically meaningful pain relief and physical HRQoL improvement was broadly consistent across stage strata and regardless of concomitant hormonal therapy, with no evidence of effect modification by stage or hormonal therapy use based on interaction testing. Robustness was further supported by multiple sensitivity analyses (complete-case restriction, exclusion of potential inflammatory confounders, additional adjustment for smoking and alcohol use, exclusion of stage IV disease, and IPTW), which yielded materially similar effect estimates (18).

From a mechanistic perspective, the observed clinical benefits of adjunctive omega-3 PUFA intake in this cohort are biologically plausible and consistent with current understanding of endometriosis as a chronic inflammatory and neuroinflammatory disorder. Endometriotic lesions are characterized by sustained activation of pro-inflammatory cytokine networks, including IL-6 and TNF-α, which promote local immune dysregulation, peripheral nociceptor sensitization, and central pain amplification (19, 20). Omega-3 PUFAs, particularly EPA and DHA, are known to modulate inflammatory signaling through multiple pathways, including competitive inhibition of arachidonic acid–derived eicosanoid synthesis, downregulation of nuclear factor-κB–mediated transcription, and generation of specialized pro-resolving mediators such as resolvins and protectins (21). These effects may attenuate cytokine production, reduce inflammatory cross-talk between immune and neural pathways, and ultimately mitigate pain perception (22). The parallel reductions in systemic IL-6, TNF-α, and CRP observed in the omega-3 PUFA group, together with improvements across multiple pain domains and physical and mental quality-of-life indices, support a model in which inflammatory modulation contributes to both symptomatic relief and functional recovery (23, 24).

When interpreted in the context of the existing literature, our findings both converge with and extend prior evidence on omega-3 PUFA supplementation in endometriosis, particularly with respect to the relationship between inflammatory modulation and clinically relevant symptom outcomes. Nodler et al. (25) conducted a double-blind, randomized, placebo-controlled trial in adolescents and young women with endometriosis and reported that fish oil supplementation did not significantly outperform placebo in reducing pelvic pain or improving quality of life, despite modest within-group improvements. In contrast, our study observed clinically meaningful pain reduction accompanied by concurrent decreases in inflammatory biomarkers and broader HRQoL gains in an adult cohort receiving omega-3 PUFA as an adjunct to standard therapy. Differences in population age, disease duration, intervention context, and outcome integration may partly account for the discrepant pain-related findings, underscoring the potential added value of adjunctive omega-3 PUFA use beyond isolated supplementation. Abokhrais et al. (26) conducted the PurFECT1 pilot randomized trial, which primarily demonstrated the feasibility and acceptability of omega-3 PUFA supplementation in women with endometriosis but was not powered to evaluate clinical efficacy and did not assess inflammatory biomarkers. Our larger cohort extends this preliminary work by providing adjusted effectiveness estimates, detailed exposure characterization, and concurrent assessment of pain, inflammatory cytokines, and multidimensional quality of life, thereby offering complementary evidence suggestive of potential clinical benefit when omega-3 PUFA is integrated into routine management. Liu et al. (11) conducted a meta-analysis of randomized controlled trials and reported no significant effects of omega-3 PUFA supplementation on pain or quality-of-life outcomes in endometriosis, while consistently demonstrating reductions in pro-inflammatory cytokines such as IL-6 and TNF-α. Our findings align with the anti-inflammatory signal observed in prior RCTs and further suggest that, in routine clinical settings, these biological effects may translate into clinically meaningful improvements in pain and HRQoL when omega-3 PUFA is used adjunctively with conventional therapy, rather than as a stand-alone intervention.

This study has several strengths. First, it adopts a clearly defined time-period–based cohort design that reflects changes in institutional practice, enabling a pragmatic comparison between conventional therapy alone and conventional therapy plus omega-3 PUFA supplementation while minimizing selection ambiguity. Second, the analysis integrates multiple clinically meaningful endpoints, including multidimensional pain assessment, systemic inflammatory biomarkers (IL-6, TNF-α, CRP), and comprehensive health-related quality-of-life measures (SF-36), within a single analytic framework, thereby enhancing internal coherence and biological plausibility. Third, omega-3 PUFA exposure was transparently characterized with respect to EPA/DHA composition, prescribed dose range, and duration, improving interpretability and clinical translatability. Fourth, the robustness of the findings was supported by extensive adjustment strategies, including multivariable modeling, subgroup analyses, sensitivity analyses, and IPTW, addressing confounding inherent to retrospective observational research. Several limitations should be considered. First, the retrospective observational design with time-period–based group allocation precludes causal inference and may be subject to secular trend confounding. Improvements in diagnostics, patient education, or adherence between 2021–2022 and 2023–2024 could have contributed to observed differences. Despite multivariable adjustment, matching, and propensity score approaches, residual confounding from unmeasured variables cannot be excluded, and findings should be interpreted as associative. Second, omega-3 PUFA exposure was identified from clinical documentation and prescription records but lacked full standardization in terms of formulation, dosing schedule, duration, and adherence, limiting dose–response inference. Third, dietary intake data were not routinely collected, precluding analysis of background nutritional patterns. This limits our ability to isolate the specific contribution of omega-3 PUFA from broader dietary influences. Fourth, while smoking and alcohol use were captured as binary baseline variables, data on exposure intensity, duration, and cumulative burden were unavailable, and potential residual confounding remains. Fifth, the relatively short follow-up duration restricted evaluation of long-term sustainability of pain relief and inflammatory modulation. Nonetheless, the consistency of findings across multiple clinical endpoints, adjusted models, subgroup analyses, and sensitivity tests, including inverse probability weighting, enhances the robustness of observed associations. Future prospective randomized trials with standardized omega-3 PUFA interventions, extended follow-up, detailed dietary and lifestyle characterization, and controlled co-interventions are warranted to confirm efficacy, define optimal patient selection, and refine dosing strategies.

## 5 Conclusion

In this retrospective cohort of patients with endometriosis, adjunctive omega-3 PUFA intake in combination with conventional therapy was associated with greater improvements in overall and domain-specific pain, larger reductions in IL-6, TNF-α, and CRP, and more favorable SF-36 PCS and MCS scores compared with conventional therapy alone. These associations remained consistent across adjusted, subgroup, and sensitivity analyses. These findings suggest that omega-3 PUFA supplementation could represent a potentially beneficial adjunctive strategy, warranting confirmation in well-designed prospective randomized trials to clarify efficacy, optimal dosing, and treatment duration.

## Data Availability

The raw data supporting the conclusions of this article will be made available by the authors, without undue reservation.
