# Supplementary material for: Omega-3 polyunsaturated fatty acid intake and pain, inflammatory cytokines, and quality of life in endometriosis
Source: Front Nutr. 2026 Mar 4;13:1768244. doi: 10.3389/fnut.2026.1768244 (PMC12996176; doi:10.3389/fnut.2026.1768244)
Supplement: Supplementary file 1 [file Table_1.docx]

Supplementary Table S1. Omega-3 PUFA exposure characteristics in the observation group

| Exposure characteristic | Overall (n = 151) | Definition / Data source |
| --- | --- | --- |
| Primary formulation used, n (%) | 138 (91.4) | Fish oil capsule; institutional formulary/drug dictionary |
| Alternative formulation used, n (%) | 13 (8.6) | Alternative omega-3 preparation with comparable EPA/DHA content; formulary |
| EPA per capsule, mg | 180 | Institutional formulary/drug dictionary |
| DHA per capsule, mg | 120 | Institutional formulary/drug dictionary |
| EPA+DHA per capsule, mg | 300 | Calculated (EPA + DHA) |
| Prescribed capsules/day, median (IQR) | 3 (2–4) | From prescription SIG |
| Prescribed capsules/day, range (min–max) | 1–6 | From prescription SIG |
| Prescribed EPA+DHA dose, mg/day, median (IQR) | 900 (600–1,200) | Calculated: (capsules/day) × 300 mg |
| Prescribed EPA+DHA dose, mg/day, range (min–max) | 300–1,800 | Calculated: (capsules/day) × 300 mg |
| Time from baseline to post-treatment assessment, weeks, median (IQR) | 12 (10–12) | Based on visit dates / documentation window |
| Time from baseline to post-treatment assessment, weeks, range (min–max) | 8–16 | Based on visit dates / documentation window |
| ≥2 omega-3 prescriptions/dispensings within follow-up window, n (%)* | 119 (78.8) | Pharmacy dispensing / repeat prescription proxy |
| Documentation of continued use at follow-up, n (%)* | 127 (84.1) | Follow-up notes / refill records |
